# Volume Overload Initiates an Immune Response in the Right Ventricle at the Neonatal Stage

**DOI:** 10.3389/fcvm.2021.772336

**Published:** 2021-11-16

**Authors:** Qing Cui, Sijuan Sun, Hongbin Zhu, Yingying Xiao, Chuan Jiang, Hao Zhang, Jinfen Liu, Lincai Ye, Jie Shen

**Affiliations:** ^1^Department of Cardiology, Shanghai Children's Medical Center, School of Medicine, Shanghai Jiao Tong University, Shanghai, China; ^2^Department of Pediatric Intensive Care Unit, Shanghai Children's Medical Center, School of Medicine, Shanghai Jiao Tong University, Shanghai, China; ^3^Department of Thoracic and Cardiovascular Surgery, Shanghai Children's Medical Center, School of Medicine, Shanghai Jiao Tong University, Shanghai, China; ^4^Shanghai Children's Medical Center, Shanghai Institute for Pediatric Congenital Heart Disease, School of Medicine, Shanghai Jiao Tong University, Shanghai, China; ^5^Shanghai Children's Medical Center, Institute of Pediatric Translational Medicine, School of Medicine, Shanghai Jiao Tong University, Shanghai, China

**Keywords:** volume overload, RNA-seq, tetralogy of Fallot, rat, right ventricular (RV)

## Abstract

**Background:** Pulmonary regurgitation caused by the correction or palliation of pediatric tetralogy of Fallot (TOF) leads to chronic right ventricular (RV) volume overload (VO), which induces adolescent RV dysfunction. A better understanding of the molecular mechanism by which VO initiates neonatal RV remodeling may bring new insights into the post-surgical management of pediatric TOF.

**Methods and Results:** We created a fistula between the abdominal aorta and inferior vena cava on postnatal day 1 (P1) using a rat model to induce neonatal VO. Echocardiography revealed that the velocity and velocity- time-integral of the pulmonary artery (PA) were significantly elevated, and hematoxylin and eosin (H&E) staining showed that the diameter of the RV significantly increased. RNA-seq analysis of the RV on P7 indicated that the top 10 enriched Gene Ontology (GO) terms and the top 20 enriched terms in the Kyoto Encyclopedia of Genes and Genomes (KEGG) pathway analysis were associated with immune responses. Flow-cytometric analysis demonstrated that the number of CD4+and CD8+ immune cells were significantly augmented in the VO group compared with the sham group.

**Conclusions:** A neonatal cardiac VO rat model on P1 was successfully created, providing a platform for studying the molecular biology of neonatal RV under the influence of VO. VO - induces an immune response at the neonatal stage (from P1 to P7), suggesting that immune responses may be an initiating factor for neonatal RV remodeling under the influence of VO and that immunosuppressants may be used to prevent pediatric RV remodeling caused by VO.

## Introduction

With the improvement of surgical technology, the survival rate of children with congenital heart disease (CHD) has significantly increased (1, 2). This in turn has led to a new generation of adolescents who may be prone to the development of heart failure due to residual lesions after correction or palliation of their cardiac defect (3, 4). The most prevalent of residual lesions after surgical correction is pulmonary regurgitation after correction for tetralogy of Fallot (TOF), leading to right ventricular (RV) volume overload (VO) (5, 6). Long-standing RV VO will induce RV dysfunction, and thus RV VO could serve as a prognostic index in the TOF population (7, 8). A better understanding the molecular mechanism by which VO initiates neonatal RV remodeling may provide new insights into the post-surgical management of pediatric TOF.

Studies using adult animal models have demonstrated that VO causes fibrosis, hypertrophy, and angiogenesis in the adult RV (9, 10). Because the neonatal heart differs from the adult heart in many aspects, the results obtained from adult hearts cannot be directly applied to neonatal hearts (11, 12). In addition, it is well-acknowledged that from postnatal day 1 (P1) to post-natal day 7 (P7), mouse cardiomyocytes (CMs) have strong proliferative and regenerative potential (13, 14). At this stage, mouse CMs are immature and utilize glycolysis as their primary source of energy (14, 15). At P7, mouse CMs begin the maturation process. At P21, the CMs are fully mature and use oxidative phosphorylation as primary energy source (12, 16). Therefore, we previously created a pre-pubertal mouse RV VO model on P7 and demonstrated that the maturation process of RV CMs is partly interrupted by VO, and the underlying mechanism is associated with the replacement of the peroxisome proliferator-activated receptor (PPAR) signaling pathway with the cell-cycle pathway (17, 18). These results highlight different responses to VO between pre-pubertal and adult RV, suggesting the importance of developmental stage-specific analysis of the effect of VO on RV remodeling.

In the present study, we constructed a neonatal RV VO model through the creation of a fistula between the abdominal aorta and inferior vena cava on P1, and then the RV free wall on P7 was selected for RNA-seq analysis to explore the possible initiating factors for pediatric RV remodeling.

## What Is New

A rat VO model was established on P1 for the first time, and we revealed how VO initiates RV responses at the neonatal stage.

## Clinical Implications

VO induces an immune response in the right ventricle at the neonatal stage, suggesting that immune responses may be an initiating factor for neonatal cardiac remodeling under the influence of VO, and immunosuppressants may be used to prevent neonatal RV remodeling.

## Materials and Methods

Methods for RNA-seq are provided in the Supplementary Materials. The bioinformatics analysis was done by the author Qing Cui with the help of Novogene Co., Ltd (Beijing, China).

### Data Availability and Ethics Statement

The data generated from this study are available from the corresponding author upon reasonable request. All RNA-seq data have been deposited in the GEO database (https://www.ncbi.nlm.nih.gov/geo) with accession number GSE180643, and primer and reagent information details are provided in Supplementary Tables 1, 2.

All procedures conformed to the principles outlined in the Declaration of Helsinki and were approved by the Animal Welfare and Human Studies Committee at Shanghai Children's Medical Center.

### Animals and Surgery

Pregnant Sprague–Dawley rats were purchased from Xipu'er-bikai Experimental Animal Co., Ltd (Shanghai, China). After birth, the rat neonates (both males and females) were randomized into two groups—, namely, a VO group and a control group on postnatal day 1 (P1)—and underwent either fistula surgery or sham operation. Briefly, after pre-anesthesia with 5% isoflurane, the neonates were transferred to an ice bed for general anesthesia, and then midline laparotomy was performed to expose the abdominal aorta (AA) and inferior vena cava (IVC). A needle (diameter: 0.07 mm) was used to puncture through the AA into the IVC (the size of the needle determined the size of the fistula). After puncture, a 2-min-hemostatic compression was executed before the abdominal wall was closed and pain was relieved with local lidocaine treatment. The neonatal rats were warmed on a heating plate until natural movements and a red/pink complexion were achieved, and then they were returned to their mother. A video is provided to better explain the surgical process (Supplementary Video 1).

### Abdominal Ultrasonography

We analyzed the fistula between the AA and IVC (AVF) and the flow at the fistula with a Vevo 2100 imaging system (Visual Sonics, Toronto, Ontario, Canada) and used a pulse-wave mode to record the waveform.

### Echocardiography

We performed echocardiography to confirm the creation of VO in the AVF rats with a Vevo 2100 imaging system (Visual Sonics). The velocity- time integral (VTI) of pulmonary artery (PA) blood flow and PA-velocity were calculated from the mean of three consecutive measurements using two-dimensional and pulsed- Doppler echocardiography.

### Histology

To further confirm VO in the RV, six rats in each group were randomly selected for morphologic examination. Following anesthetization with 5% isoflurane, rat hearts were removed, fixed in 10% paraformaldehyde overnight at room temperature, dehydrated in an ethanol series, embedded in paraffin, and 6-μm-thick sections were prepared. The sections were stained with hematoxylin and eosin (H&E) kits (Beyotime biotech, Shanghai, China) according to the manufacturer's instructions.

### Immunofluorescence

Frozen RVs were sectioned to a thickness of 8-μm, washed thrice with phosphate-buffered saline (PBS), fixed with 4% paraformaldehyde for 10 min, permeated with 0.5% Triton X-100 for 15 min, blocked with 10% donkey serum for 30 min, and stained with primary antibodies overnight at 4°C. After washing the slides three more times, we incubated the sections or cells with secondary antibodies and 4′,6-diamidino-2-phenylindole (DAPI) for 30 min. Three researchers who were blinded to the samples performed the quantification.

### Flow Cytometry, Cell Sorting, and Analysis

To evaluate immune cell infiltrates in the postnatal heart, heart tissues were minced into small fragments and dissociated with 1:1 type II collagenase (1,000 U/mL in PBS, Worthington, Lakewood, NJ, USA) and dispase (11 U/mL in PBS, Gibco, Waltham, MA, USA) at 37°C for 30 min. Enzymatic action was halted by adding 10% FBS and the dissociated cells were washed twice with PBS. The dissociated cardiac cells were removed from the contaminating erythrocytes by incubation with red blood cell lysis buffer (eBiosciences, Waltham, MA, USA) for 5 min and then blocked with 2% normal rabbit serum. Cells were subsequently stained with fluorochrome-conjugated antibodies against CD4 and CD8 (Biolegend or eBiosciences) at a dilution of 1:100 at 4°C for 30 min. The cells were then washed thrice with 2% FBS-containing PBS and analyzed on a flow cytometer (BD, FACSAria™ Fusion). Propidium iodide (PI, BD)- positive dead cells were excluded for live cell analysis/sorting, and FACS data were then analyzed with FlowJo software.

### Statistical Analysis

We performed statistical analyses using SAS software, ver. 9.2 (SAS Institute Inc., Cary, NC, USA). Continuous data were expressed as means ± one standard deviation. Data differences were analyzed with the student's *t*-test when the data were normally distributed; otherwise, we used the rank-sum test. Differences with *P*-values < 0.05 were considered statistically significant.

## Results

### Generation of RV VO in AVF Rats

**Figure 1:**
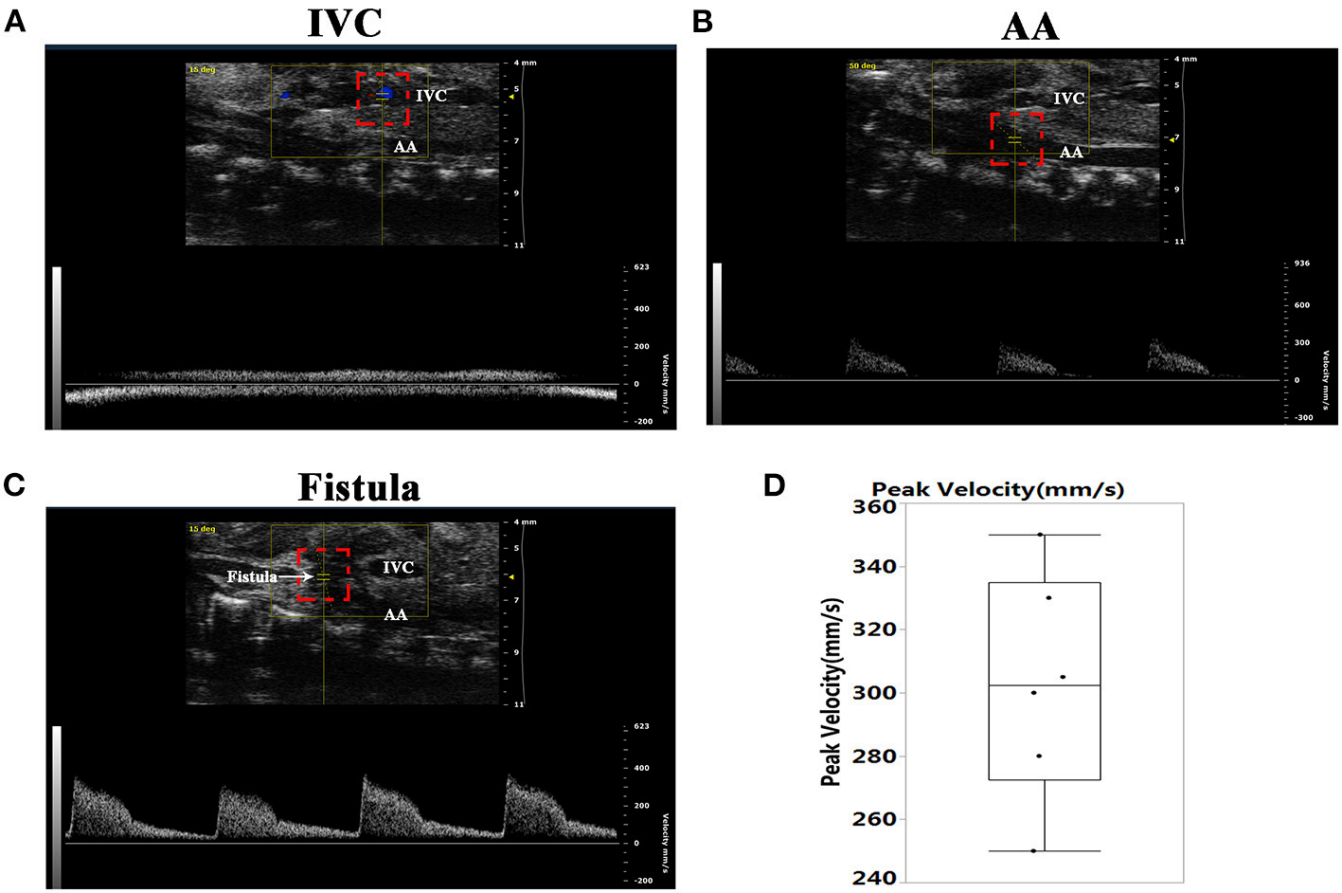
Establishment of the abdominal aorta and inferior vena cava fistula (AVF). **(A)** The inferior vena cava (IVC) manifested no pulsatile blood flow. **(B)** There was pulsatile blood flow in the abdominal aorta (AA), with a peak blood flow velocity of 300 mm/s. **(C)** Representative image of the pulsating blood flow at the fistula, with a peak blood flow velocity of 400 mm/s. **(D)** Quantification of peak velocity at the fistula.

As shown in Figure 1A, there was no pulsatile blood flow in the inferior vena cava (IVC). However, a pulsatile blood flow appeared in the abdominal aorta (AA), with a peak flow velocity up to 300 mm/s (Figure 1B). At the puncture point (PP), we noted a pulsatile blood flow with a peak flow velocity up to 400 mm/s (Figure 1C), and the mean peak velocity in the fistula was 302.5 ± 35.5 (Figure 1D). These results suggested that a fistula between the AA and IVC was successfully created.

**Figure 2:**
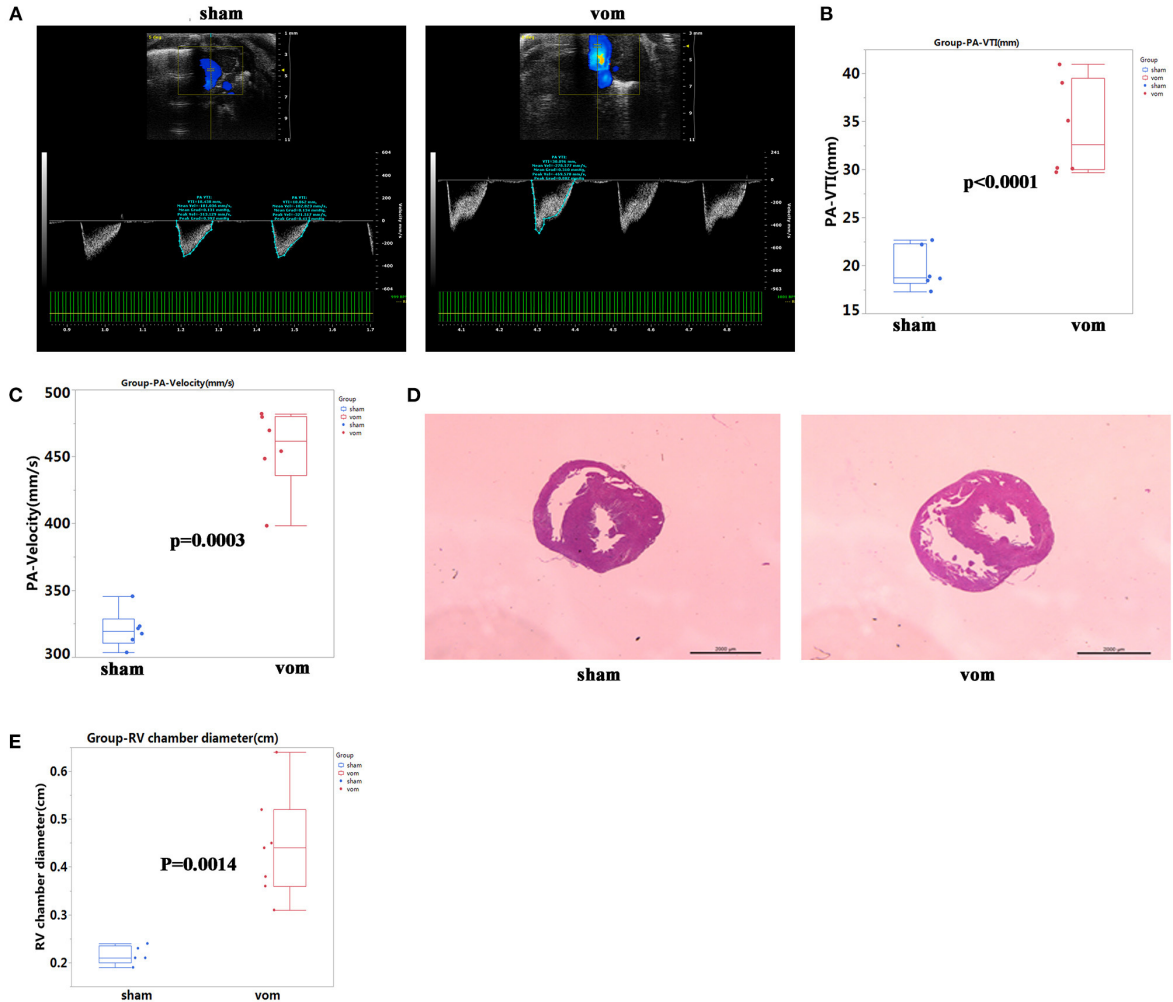
Verification of VO in the AVF group. **(A)** Representative echocardiogram of pulmonary artery (PA) velocity time integral (VTI) and velocity in the sham and VO model (vom) groups. **(B)** Quantification of PA- VTI in the sham and vom groups, *N* = 6. **(C)** Quantification of PA-velocity in the sham and vom groups, *N* = 6. **(D)** Middle panel, representative H&E staining of hearts from the sham and vom groups. **(E)** Middle panel, quantification of the diameter of the RV chamber.

To confirm that there was VO in the RV, we examined the pulmonary artery (PA)-velocity time integral (VTI) and PA-velocity on P7. The PA-VTIs in the sham and VO groups were 19.7 ± 2.2 and 34.2 ± 5.0 mm, respectively (*P* < 0.0001; *n* = 6; Figures 2A,B), and the PA- velocities in the sham and VO groups were 320.8 ± 14.0 and 455.3 ± 31.0 mm/s, respectively (*P* < 0.0001; *n* = 6; Figures 2A,C). H&E staining also showed that the diameter of the RV chamber at the middle panel in the VO group significantly increased (Figures 2D,E). These results thus verified that RV VO was successfully implemented.

### Overview of Differentially Expressed Genes in the RV Between VO and Sham Groups

**Figure 3:**
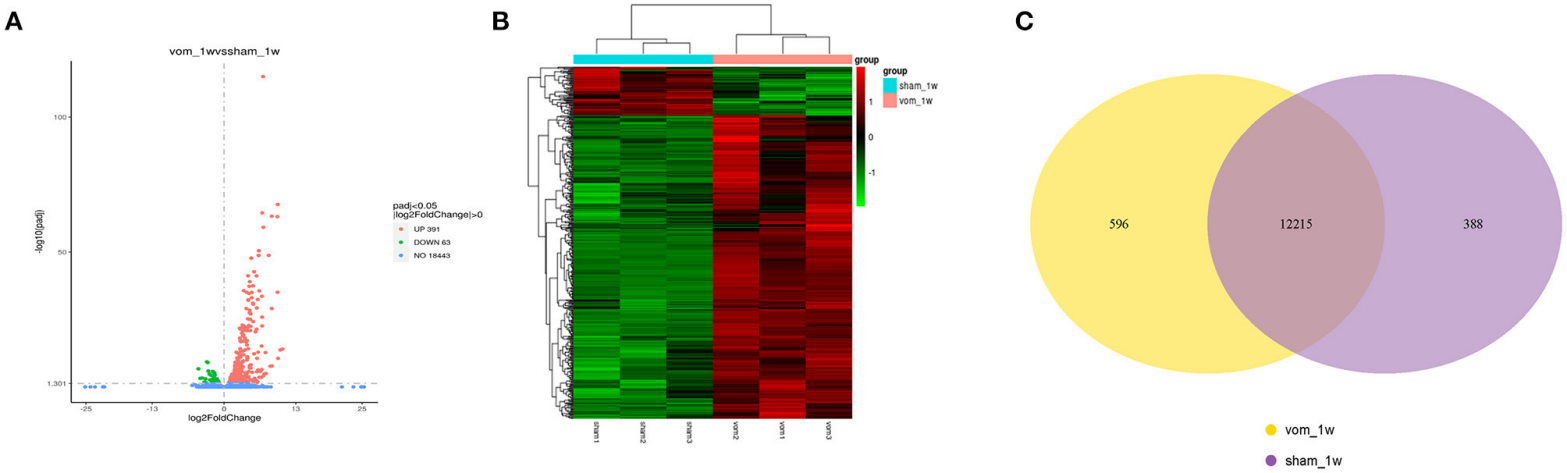
The transcriptome of the neonatal RV is altered by VO. **(A)** Volcano map of the differentially expressed genes between the vom and sham groups. There were 391 upregulated and 63 downregulated genes in the vom group. **(B)** Cluster analysis of the differentially expressed genes between the vom and sham groups. The clusters of genes in each group were quite different from each other but were similar within the same group. **(C)** Venn diagram of the differentially expressed genes between the vom and sham groups. These two groups shared 12,215 commonly expressed genes; there were 596 genes expressed only in the vom group and 388 genes expressed only in the sham group.

To understand how VO induced transcriptome alterations in the neonatal RV, we performed RNA-seq analysis of the RV free wall. As shown in Figure 3A, there were 454 differentially expressed genes between the two groups, of which 63 were downregulated and 391 were upregulated. When these genes were clustered, a heat map showed that the individual rats in the same group were similar to each other, but they markedly differed from the other group (Figure 3B). These two groups shared 11,215 expressed genes. In the VO group, another 596 genes were expressed (VO model_1week, vom_1w), and 388 genes were expressed only in the sham group (sham1 week, sham_1w) (Figure 3C).

### Most Enriched GO Terms Are Associated With Immune Response

**Figure 4:**
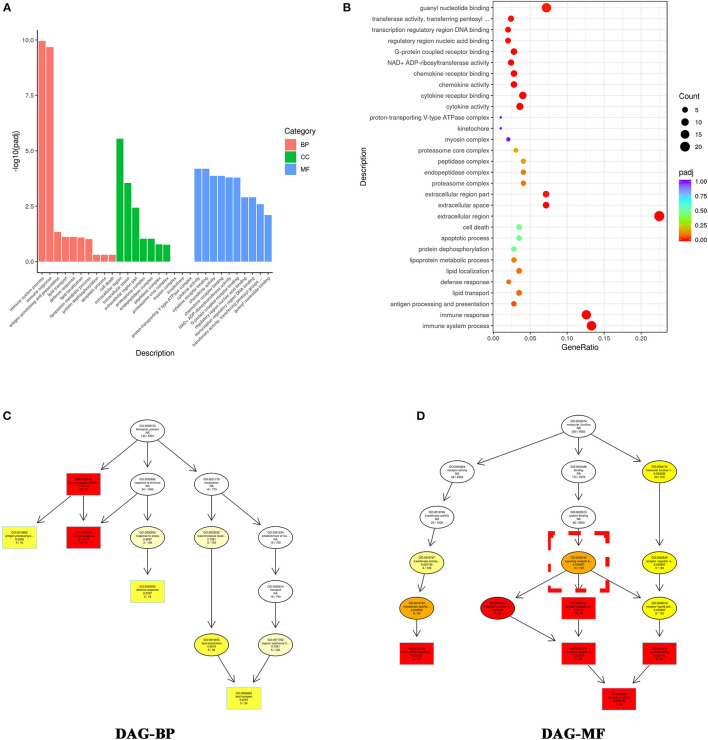
GO enrichment analysis indicates that the differentially expressed genes altered by VO are primarily associated with immune response. **(A)** Histogram of the GO analysis. From the results of the GO enrichment analysis, we displayed the 10 most significant terms. The abscissa is the GO -term, and the ordinate is the significance level for GO term enrichment; the higher the value, the more significant the result. The different colors represent three different GO subclasses: biological process (BP), cellular component (CC), and molecular function (MF). **(B)** Scatter plots of GO analysis. From the results of the GO-enrichment analysis, we selected the 30 most significant terms to construct scatter plots for display. The size of the dots represents the number of genes annotated to the GO term, and the colors from red to purple represent the significance level of the GO term enrichment. **(C)** DAG analysis of biological process (BP). **(D)** DAG analysis of molecular function (MF). The intensity of the color represents the degree of enrichment; a darker color indicates a higher degree of enrichment. Each node displays the name of the term and the adjusted *P*-value (padj).

We applied Gene Ontology (GO) analysis to assess differentially expressed genes. As shown in Figures 4A,B, the top 10 most enriched GO terms in the biological process (BP) functional category included immune system process, immune response, antigen processing and presentation, lipid transport, defense response, lipid localization, lipoprotein metabolic process, protein dephosphorylation, apoptotic process, and cell death. Cell component (CC) analysis showed the top 10 most enriched GO terms were extracellular region, extracellular space, extracellular region part, proteasome complex, endopeptidase complex, proteasome core complex, myosin complex, kinetochore, and proton-transporting V-type ATPase complex (Figures 4A,B). Molecular function (MF) analysis showed the top four most enriched GO terms were cytokine activity, cytokine receptor binding, chemokine activity, and chemokine receptor binding (Figures 4A,B). These results suggested that VO caused cell death, and that cell death stimulated an immune response, with the latter including cytokines and the interaction between their receptors.

To further understand the relationship between the most enriched GO terms, we created a directed acyclic graph (DAG). In the DAG, the top five enriched GO terms were plotted as master nodes, and through the inclusion relationship, the related GO terms were displayed together. The results showed that signaling receptor binding (GO:0005102) included the top four most enriched GO terms (Figures 4C,D), indicating that signaling receptor binding may play a critical role in VO- induced immune response.

### KEGG-Pathway Analysis Shows That Most Enriched Terms Are Associated With Immune Response

**Figure 5:**
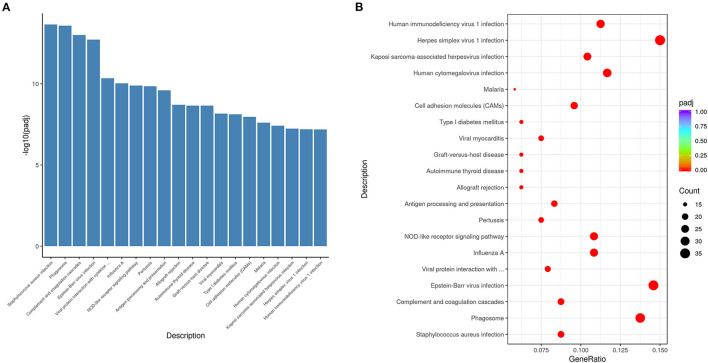
KEGG pathway analysis indicates that the differentially expressed genes altered by VO are principally associated with immune response. **(A)** Histogram of the 20 most significant KEGG pathways. The 20 most significant KEGG pathways from the KEGG enrichment results are displayed. The abscissa is the KEGG pathway, and the ordinate is the significance level of pathway enrichment; the higher the value, the greater the significance. **(B)** Scatterplot of the 20 most significant KEGG pathways. The abscissa is the ratio of the number of genes in the KEGG pathway analysis to the total number of differentially expressed genes, the ordinate is the KEGG pathway, the size of the dots represents the number of genes annotated to the KEGG pathway, and the colors from red to purple represent the significance level of KEGG pathway enrichment.

We performed KEGG- pathway analysis to uncover whether the pathways regulating differentially expressed genes were associated with immune responses. The top 20 enriched pathways were *Staphylococcus aureus* infection, phagosome, complement and coagulation cascades, Epstein-Barr virus infection, viral protein interaction with cytokine, influenza A, NOD-like receptor signaling pathway, pertussis, antigen processing and presentation, allograft rejection, autoimmune thyroid disease, graft-vs.-host disease, viral myocarditis, type I diabetes mellitus, malaria, human cytomegalovirus infection, Kaposi sarcoma-associated herpesvirus infection, herpes simplex virus 1 infection, and human immunodeficiency virus 1 infection (Figures 5A,B). These results suggested that immune response was the major process activated by VO at the neonatal stage.

### Verification of RNA-Seq Results by the Examination of Immune Cells

To confirm the RNA-seq results, we verified the 20 genes that manifested the greatest fold-changes by quantitative real-time PCR (qRT-PCR). We further analyzed the percentage of immune cells in the VO and sham groups.

**Figure 6:**
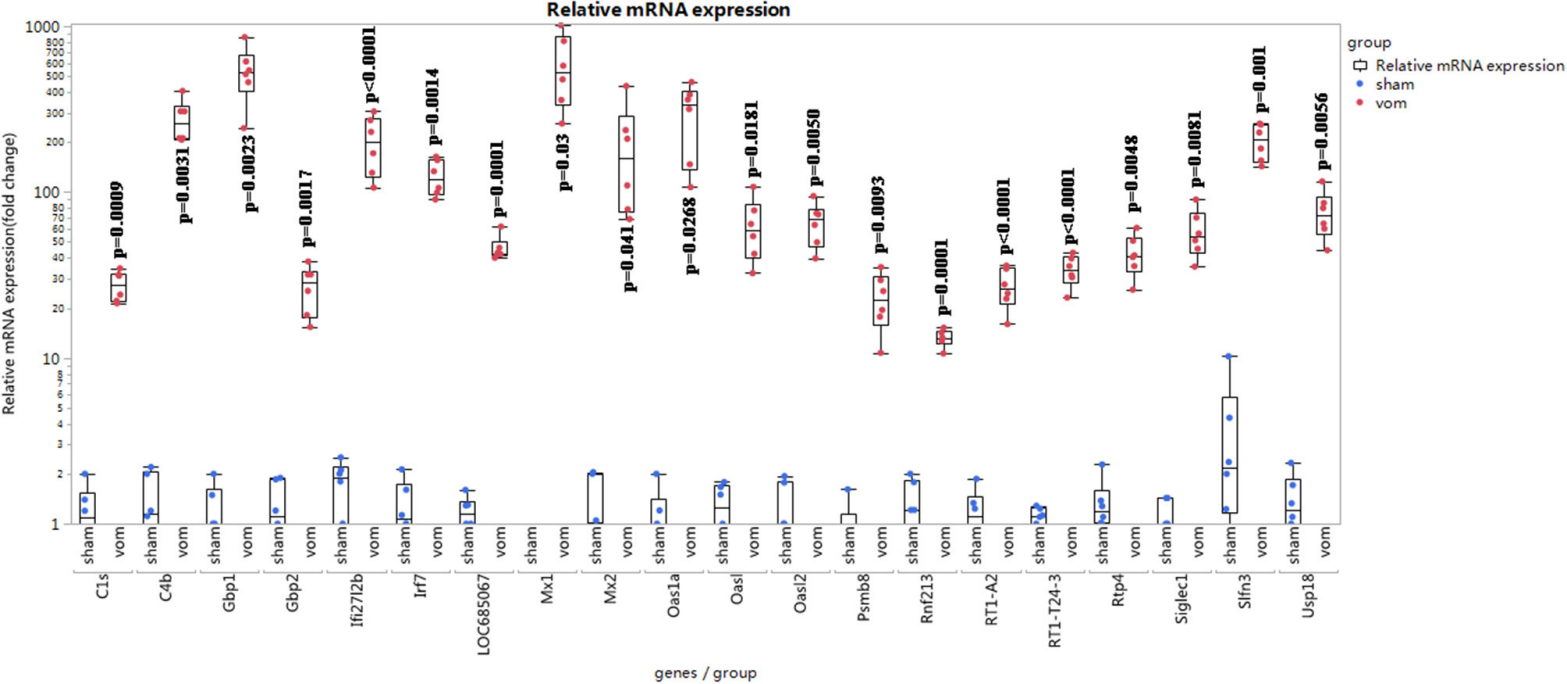
Relative mRNA expression of top 20 differentially expressed genes in the RNA-seq data. The relative fold change was calculated using the ΔΔCT method (19). One sample in the sham group was set as “1”; the other samples referred to the sample.

**Figure 7:**
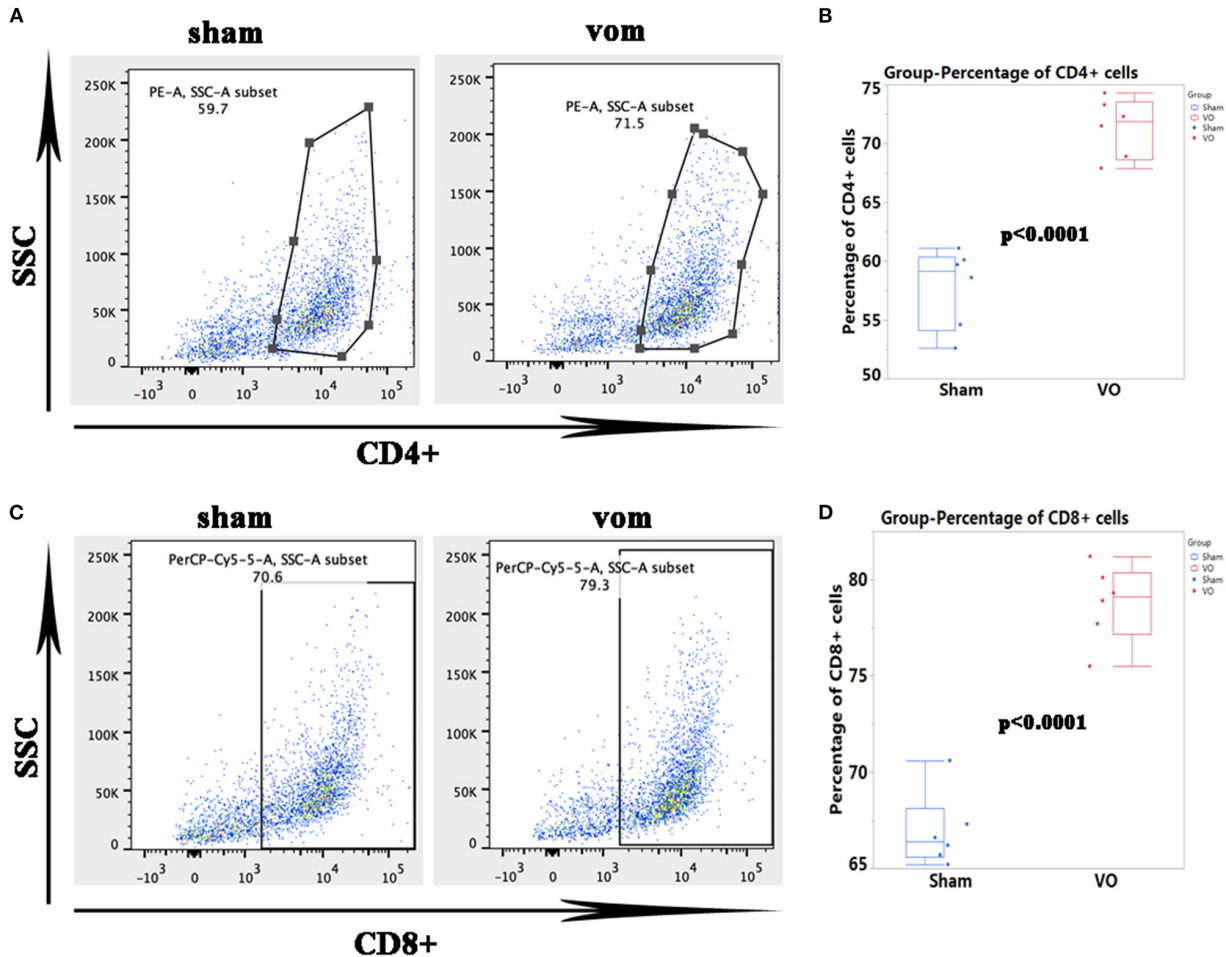
Verification of the RNA-seq data by flow cytometric analysis of immune cells. **(A)** Representative dots of CD4+ cells. **(B)** Quantification of the percentage of CD4+ cells. **(C)** Representative dots of CD8+ immune cells. **(D)** Quantification of the percentage of CD8+ cells.

As shown in Figure 6, for the top 20 genes from the RNA-seq data, 18 of them (*Irf7, Mx1, Slfn3, C4b, Gbp1, Ifi27l2b, Usp18, Mx2, Oasl2, RT1-T24-3, LOC685067, RT1-A2, Siglec1, C1s, Rtp4, Gbp2, Oasl, and Oas1a*) were closely related to immune responses. As shown in Figures 7A,B, the percentages of CD4+ cells in the sham and VO groups were 57.8 ± 3.4 and 71.4 ± 2.5% (*p* < 0.0001), respectively, whereas the percentages of CD8+ cells in the sham and VO groups were 66.9 ± 1.9 and 78.7 ± 2.0% (*p* < 0.0001), respectively (Figures 7C,D). These results confirmed that the immune response was activated by VO at the neonatal stage.

Because CM proliferation is one of the main features at the neonatal stage, we also examined proliferation markers. As shown in Supplementary Figure 1, quantified amounts of the cell-cycle markers- Ki67 and pHH3 were comparable between the sham and VO groups, suggesting that the cell cycle was not activated by VO at the neonatal stage.

## Discussion

TOF is the most common cyanotic congenital heart disease (20). One of its main features is obstruction of the RV outflow tract, which is usually enlarged with a transvalvular patch to relieve the obstruction during surgical correction (21). This kind of surgical method generally causes pulmonary regurgitation (22). Pulmonary regurgitation producing chronic VO is the most common pathway that ultimately leads to RV dysfunction and cardiovascular mortality in patients with TOF (23). However, the molecular mechanisms initiating and driving RV dysfunction under the influence of VO remains unclear. Systematic reviews have highlighted the translational gap from bench to bedside in terms of RV VO animal models and reveals that there is lack of neonatal RV VO models (10, 17, 18). The current study introduced a neonatal rat RV VO model to study pediatric RV VO, which may narrow down the translational gap from bench to bedside.

In the adulthood, VO causes fibrosis, hypertrophy, and angiogenesis (9, 10); additionally, in the pre-pubetal stage, VO interrupts cardiomyocyte maturation and reactive cell cycle (17, 18). Current data show that at the neonatal stage, VO induces immune responses, suggesting that immune responses may be an initiating factor for neonatal cardiac remodeling under the influence of VO and immunosuppressants may be used to prevent neonatal RV remodeling, ultimately leading to RV dysfunction.

There are two reasons why a neonatal rat VO model is needed when there is a pre-pubetal mouse VO model (17, 18). The physiological characteristics of the heart between the pre-pubetal and neonatal period are different. At the neonatal stage, rodent CMs are immature and use glycolysis as their primary energy source, with a much stronger proliferative and regenerative potential than pre-pubetal CMs (11–13). Second, there are much more surgical spaces for the operation in neonatal rat than in mouse, with a much higher survival rate in rat than in mouse in our experience. To learn the skill, neonatal rats may be the first choice. The current study also highlights the developmental stage-specific influence of VO on RV remodeling.

Whether immune response is the initiating factor that stimulates pediatric cardiac remodeling under the influence of VO needs to be verified with corresponding human cardiac samples. We have considered cardiac specimens from pediatric TOF patients, but the results obtained from TOF are contaminated by pressure overload. It is possible that comparing the heart functions or myocardial specimens of children with surgical-repaired TOF with immunosuppressive agents and those without immunosuppressive agents can provide evidence for this conclusion. It is essential to elucidate the underlying mechanisms for the elevated immune responses. As shown in our current data (Figures 4A,B), cell death and apoptotic process were among the top 10 enriched GO terms. It is possible that cell death caused by VO activates an immune response. Second, a recent study demonstrated that immune responses such as signals lymphoangiocrine and regulatory T cells play an important role in neonatal cardiac regeneration and repair (24, 25). It is possible that moderate immune responses or paracrine signals from lymphatics and regulatory T cells are helpful in the neonatal RV's response to VO. In addition, in our previous pre-pubetal VO study, immunosuppressant (Cyclosporine, CsA) partly restored VO-induced changes in gene expression (Supplementary Figure 2). All these results suggest that immune response plays a critical role in the VO-induced RV remodeling at the neonatal stage.

Another important concern is that the molecular differences between shunt-VO and pulmonary regurgitation -VO models. To what extent can AVF-shunt-VO simulate pulmonary regurgitation-VO, which is the case of repaired TOF? A systematic review demonstrated that in adult animals, there were no notably different hemodynamic responses and RV remodeling between shunt-VO and pulmonary regurgitation -VO models (10). However, due to differences between young and adult RVs, the results obtained from the review could not be directly applied to young RV, and the young pulmonary regurgitation-VO model is required to explore whether there are molecular differences between the shunt-VO and pulmonary regurgitation-VO models. In addition, due to the limited space for pulmonary regurgitation-surgery in neonatal rats, using large animals to perform pulmonary regurgitation-surgery may be a more practical approach.

In summary, the current study is the first to introduce a neonatal RV VO rat model, providing a platform to narrow down the translational gap from bench to bedside. Additionally, we revealed that immune response may be a factor initiating pediatric cardiac RV remodeling under the influence of VO, suggesting that the application of immunosuppressive agents may be an alternative method to manage pediatric TOF after surgical correction.

## Data Availability Statement

The datasets presented in this study can be found in online repositories. The names of the repository/repositories and accession number(s) can be found in the article/Supplementary Material.

## Ethics Statement

The animal study was reviewed and approved by the Animal Welfare and Human Studies Committee at Shanghai Children's Medical Center.

## Author Contributions

QC, SS, LY, and JS designed the study. QC and SS performed the experiments. CJ collected the samples. QC, SS, and LY conducted the statistical analysis. QC, LY, HZ, and JL wrote the manuscript. JS and LY reviewed and edited the manuscript. All authors read and approved the final manuscript.

## Funding

This work was supported by the National Key R&D Program of China (No. 2019YFA0110401), the Science and Technology Innovation Action Plan of Shanghai—Experimental Animal Research (No. 201409005900), the National Natural Science Foundation of China (No. 81800285), and the Foundation of Pudong Science and Technology Development (No. PKJ2019-Y12).

## Conflict of Interest

The authors declare that the research was conducted in the absence of any commercial or financial relationships that could be construed as a potential conflict of interest.

## Publisher's Note

All claims expressed in this article are solely those of the authors and do not necessarily represent those of their affiliated organizations, or those of the publisher, the editors and the reviewers. Any product that may be evaluated in this article, or claim that may be made by its manufacturer, is not guaranteed or endorsed by the publisher.

## Supplementary Material

Supplementary Figure 1 Proliferation markers in the cardiomyocytes are unchanged by VO. **(A)** Representative Ki67-positive cardiomyocytes in the sham and vom groups. Arrow indicates Ki67-positive cardiomyocytes, star indicates Ki67-positive non-cardiomyocytes. **(B)** Quantification of Ki67-positive cardiomyocytes (*N* = six mice). The arrow indicates pHH3-positive cardiomyocytes, the asterisk indicates pHH3-positive non-cardiomyocytes. **(C)** Representative pHH3-positive cardiomyocytes in the sham and vom groups. **(D)** Quantification of the pHH3-positive cardiomyocytes (*N* = 6 mice).

Supplementary Figure 2 Cyclosporine (CsA) partly restored VO-induced changes in gene expression at the pre-pubetal stage.

Supplementary Table 1 Primer information.

Supplementary Table 2 Reagents.

Supplementary video 1 Surgical procedures for establishment of the neonatal rat AVF model.
