# Supplementary figures and images for: Volume Overload Initiates an Immune Response in the Right Ventricle at the Neonatal Stage
Source: Front Cardiovasc Med. 2021 Nov 16;8:772336. doi: 10.3389/fcvm.2021.772336 (PMC8635051; doi:10.3389/fcvm.2021.772336)

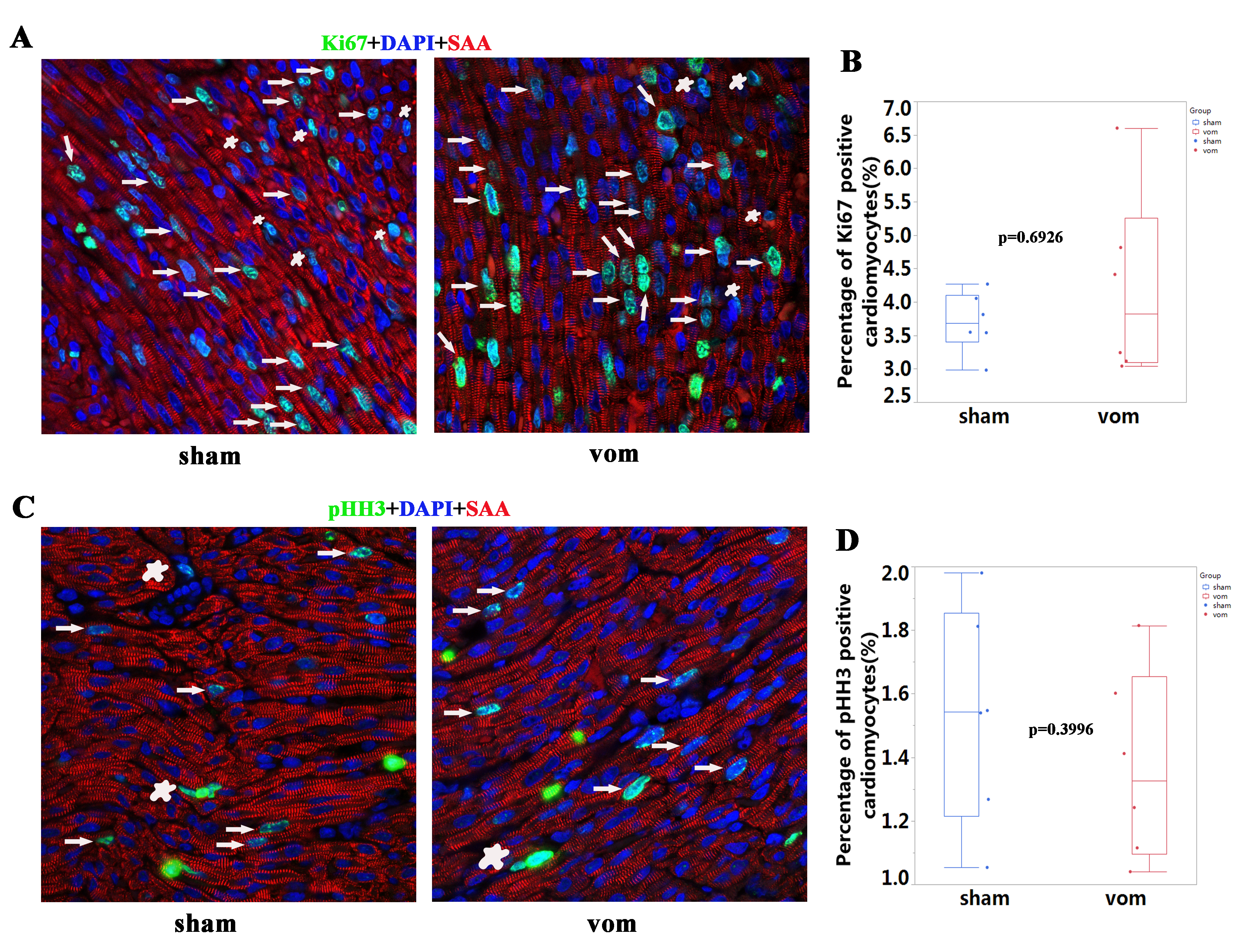

Supplement: Supplementary Figure 1 — Proliferation markers in the cardiomyocytes are unchanged by VO. (A) Representative Ki67-positive cardiomyocytes in the sham and vom groups. Arrow indicates Ki67-positive cardiomyocytes, star indicates Ki67-positive non-cardiomyocytes. (B) Quantification of Ki67-positive cardiomyocytes (N = six mice). The arrow indicates pHH3-positive cardiomyocytes, the asterisk indicates pHH3-positive non-cardiomyocytes. (C) Representative pHH3-positive cardiomyocytes in the sham and vom groups. (D) Quantification of the pHH3-positive cardiomyocytes (N = 6 mice). [file Image_1.TIF]

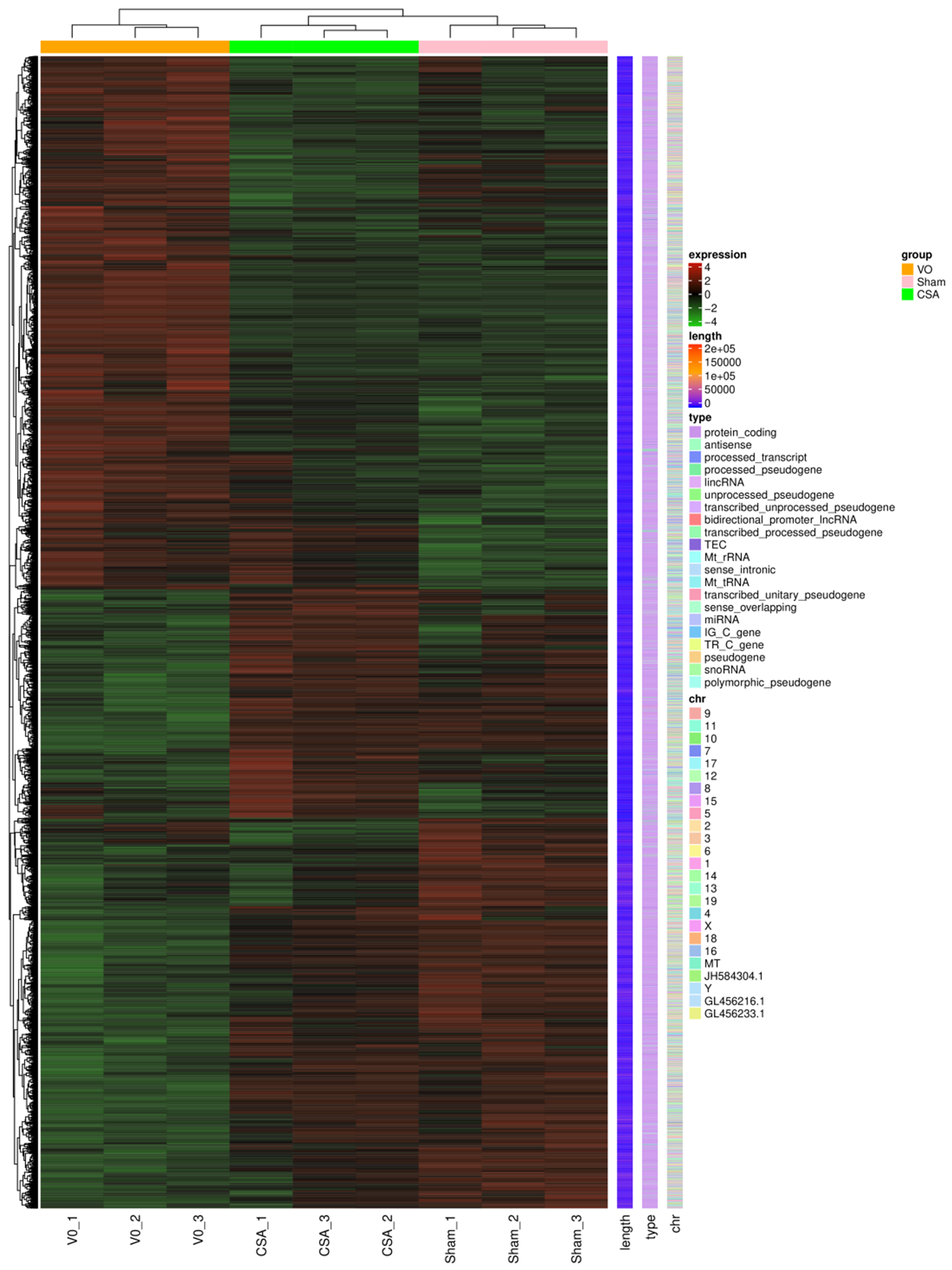

Supplement: Supplementary Figure 2 — Cyclosporine (CsA) partly restored VO-induced changes in gene expression at the pre-pubetal stage. [file Image_2.PNG]
