# Supplementary material for: Volume Overload Initiates an Immune Response in the Right Ventricle at the Neonatal Stage
Source: Front Cardiovasc Med. 2021 Nov 16;8:772336. doi: 10.3389/fcvm.2021.772336 (PMC8635051; doi:10.3389/fcvm.2021.772336)
Supplement: Supplementary Table 1 — Primer information. [file Table_1.DOCX]

Supplemental Table 1 Primer information

| Gene |  | Sequence (5'->3') |
| --- | --- | --- |
| Irf7 | Forward | TTCAGCCGTAGGGATCTGGA |
|  | Reverse | ATCACCAGAAAGCAGAGGGC |
| Mx1 | Forward | CCACCTGGAAAACACGCAAG |
|  | Reverse | CCATTGCCTGGCTCTCTTCA |
| Slfn3 | Forward | AGTGAAGAGTGGCTGTCACC |
|  | Reverse | TCACTGAGCCGTTGAATCTTTT |
| C4b | Forward | TGGACGATGGCTCATTCCAC |
|  | Reverse | TGCCACTGTCTCGTCGGT |
| Gbp1 | Forward | TGATGTCAATCCCACTCCCT |
|  | Reverse | GGTAGAGGCCCACTATTGCC |
| Ifi27l2b | Forward | TGTGGGGAGAAATGTTGGGC |
|  | Reverse | CAGACATCATCTTGGCCGCT |
| Usp18 | Forward | TGTGGAATCCTGACCTCCAG |
|  | Reverse | TCCCAACATGGGCAATCACA |
| Mx2 | Forward | AGGGAGAAAGAATGTCGCCT |
|  | Reverse | AGGCAGCCCGTACAATTTCA |
| Oasl2 | Forward | GCTCCTACCCAAAGGTTCCC |
|  | Reverse | TCTCCCGCTGAAGCTACTCT |
| RT1-T24-3 | Forward | GGACTTCAAGCAACCACCCT |
|  | Reverse | TCCTCTTCAGGAACTGACGGA |
| LOC685067 | Forward | GGAATCCGGGCTGCTTTATG |
|  | Reverse | TGAAAGTCGGAGGTTTGCACT |
| RT1-A2 | Forward | GCGATGCAGAGAATCCGAGA |
|  | Reverse | ATGATGGCTCCAAGGACGAC |
| Siglec1 | Forward | TCCATACCCCAAGTGTTGCC |
|  | Reverse | TCCCAGGACACCAATTCAGAC |
| C1s | Forward | CTCGAAGATCAAGGCCGGTT |
|  | Reverse | AGCTGATGGGAACAAGTGGG |
| Rtp4 | Forward | CTTGACTTTCCAGCCTGCCA |
|  | Reverse | GGGGGAAGCATTACCCAGTC |
| Psmb8 | Forward | ATGACAATGGGACTCGGCTC |
|  | Reverse | CGGTACCCACTGTCCATCAC |
| Gbp2 | Forward | AGTACCTGGAGCATTCGCTG |
|  | Reverse | ATGCACAGTCGAGGCTCATT |
| Rnf213 | Forward | GGACCACCCACGTGAGTAAA |
|  | Reverse | TCTTAGGGCCCTTGTCCACT |
| Oasl | Forward | GCCTCCTACGGTTGGTCAAA |
|  | Reverse | CATGACAGTGGCGAGACCTT |
| Oas1a | Forward | GAAGTGCCGGTGGATGAGG |
|  | Reverse | CCGCCCCTTCTGAATCTGTT |
