# Supplementary material for: Volume Overload Initiates an Immune Response in the Right Ventricle at the Neonatal Stage
Source: Front Cardiovasc Med. 2021 Nov 16;8:772336. doi: 10.3389/fcvm.2021.772336 (PMC8635051; doi:10.3389/fcvm.2021.772336)
Supplement: Supplementary Table 2 — Reagents. [file Table_2.DOCX]

Supplemental Table 2 Reagents

| Name | Company | Catalog No. |
| --- | --- | --- |
| Triton X-100 | Sigma-Aldrich | T9284 |
| Hematoxylin and eosin Kit | Beyotime biotech | C0105M |
| DNase | Worthington, Lakewood, NJ, USA | 9003-98-9 |
| Type II collagenase | Worthington | 9001-12-1 |
| Dispase | Gibco | 17105041 |
| RNase | Worthington, Lakewood, NJ, USA | 9001-99-4 |
| paraformaldehyde(PFA) | Sigma-Aldrich | 158127 |
| 4',6-diamidino-2-phenylindole(DAPI) | ThermoFisher Scientific | D3571 |
| pH3 antibody | Milipore | 06-570 |
| Ki67 antibody | Abcam | ab15580 |
| sarcomeric α-actinin antibody | Abcam | ab9465 |
| PureLink RNA Micro Scale Kit | Life Technologies, Carlsbad, California, USA | 12183016 |
| PrimeScriptTM reagent kit | Takara Bio, Kusatsu, Japan | RR037A |
| SYBR Green Power Premix Kits | Applied Biosystems, Foster City, California | 4368577 |
| NEB Next® UltraTM RNA Library Prep Kit | NEB, USA | E7760 |
| CD45-APC-Cy7 antibody | BD Pharmingen | 561586 |
| CD8-Percp-cy5.5 antibody | BD Pharmingen | 555368 |
| CD4-PE antibody | BD Pharmingen | 550628 |
| TruSeq PE Cluster Kit | Illumina | v3-cBot-HS |
