# Supplementary material for: Volume Overload Initiates an Immune Response in the Right Ventricle at the Neonatal Stage
Source: Front Cardiovasc Med. 2021 Nov 16;8:772336. doi: 10.3389/fcvm.2021.772336 (PMC8635051; doi:10.3389/fcvm.2021.772336)
Supplement: Supplementary file 6 [file Data_Sheet_1.DOCX]

**Methods for RNA-seq**

**RNA quantification and qualification of the RV free wall**

After anesthetizing the rats with 5% isoflurane, their thoracic cavities were opened to obtain the RV free wall, which was used for RNA extraction with a PureLink RNA Micro Scale Kit. RNA degradation and contamination were monitored on 1% agarose gels, and RNA purity was assessed using a NanoPhotometer^®^spectrophotometer (IMPLEN, CA, USA). RNA integrity was evaluated using the RNA Nano 6000 assay Kit of the Bioanalyzer 2100 system (Agilent Technologies, CA, USA). We executed RT-PCR with a PrimeScript reagent kit and performed qRT-PCR using SYBR Green Power Premix Kit, according to the manufacturers’ instructions and with a 7900 Fast Real-Time PCR System (Applied Biosystems). The following PCR cycle conditions were used: 1 cycle at 95°C for 10 s, followed by 40 cycles of 95°C for 15 s and 60°C for 60 s. The primers were obtained from Generay Biotech Co. Ltd. (Shanghai, China). The relative fold change was calculated using the ΔΔCt method.

**Library preparation**

A total of 1 μg of RNA per sample from the RV free wall was used as the input material for RNA sample preparation, and sequencing libraries were generated using the NEBNext^®^ Ultra^TM^ RNA Library Prep Kit for Illumina^®^ (NEB, USA) according to the manufacturer’s instructions. Index codes were added to attribute sequences to each sample. Briefly, mRNA was purified from total RNA using poly-T oligo-attached magnetic beads. Fragmentation was carried out using divalent cations under an elevated temperature in a NEB Next First Strand Synthesis Reaction Buffer (5×), and first-strand cDNA was synthesized using random hexamer primers and M-MuLV Reverse Transcriptase (RNase H ^–^); second-strand cDNA synthesis was subsequently performed using DNA polymerase I and RNase H. Remaining overhangs were converted into blunt ends via exonuclease/polymerase activities, and after adenylation of the 3’ ends of the DNA fragments, NEBNext Adaptors with hairpin loop structures were ligated to prepare the sequences for hybridization. To select cDNA fragments that were preferentially 250–300 bp in length, the library fragments were purified with an AMPure XP system (Beckman Coulter, Beverly, MA, USA). Three microliters of USER Enzyme (NEB, USA) was used with size-selected, adaptor-ligated cDNA at 37°C for 15 min followed by 5 min at 95°C. Then, PCR was performed with Phusion High-Fidelity DNA polymerase, Universal PCR primers, and Index (X) Primer. PCR products were ultimately purified (AMPure XP system), and library quality was assessed on an Agilent Bioanalyzer 2100 system.

**Clustering and sequencing**

The clustering of the index-coded samples was performed on a cBot Cluster Generation System using a TruSeq PE Cluster Kit v3-cBot-HS (Illumina) according to the manufacturer’s instructions. Sequencing was then performed on an Illumina Novaseq platform to generate 150 -bp paired-end reads.

**Quality control, read mapping, and quantification of gene expression levels**

Raw data (raw reads) in fastq format were first processed through in-house Perl scripts; and reads containing adapters, reads containing poly-N, and low quality reads were removed from the raw data to generate clean data (clean reads). All of the downstream analyses were thus based on clean, high-quality data.

The reference genome and gene model annotation files were downloaded from the genome website directly. The index of the reference genome was constructed using Hisat2 v2.0.5, and paired-end clean reads were also aligned to the reference genome using Hisat2 v2.0.5; the number of reads mapped to each gene was counted using featureCounts v1.5.0-p3. The fragments per kilobase of transcript sequence per million base pairs sequenced (FPKM) for each gene was calculated based on the length of the gene and read counts mapped to each gene.

**Differential gene expression analysis**

Differential gene expression analysis was performed using the DESeq2 R package (1.16.1). DESeq2 provides statistical routines for determining the downregulated expression in digital gene expression data using a model based on a negative binomial distribution. The resulting P-values were adjusted using Benjamini and Hochberg’s approach for controlling the false discovery rate. Genes with an adjusted *P*-value of < 0.05 as determined by DESeq2 were considered to have downregulated expression.

**Gene Ontology (GO) and KEGG enrichment analyses**

GO enrichment was implemented with the clusterProfiler R package. GO terms with corrected *P* values under 0.05 were considered to be significantly enriched, and the clusterProfiler R package was used to test the statistical enrichment of genes that were downregulated in KEGG pathways (http://www.genome.jp/kegg/).
